# The Investigation of England's Vitality

**Published:** 1915-03-06

**Authors:** 


### THE INVESTIGATION OF ENGLAND'S VITALITY.

### Actual Results of Public Health Work.

Owing to the war, immense importance attaches to the condition of the national vitality, and when the war is over we may expect great attention to be paid, for purposes of comparison, to the state of the nation's vigour immediately preceding it. All interested in public health administration will, therefore, study with lively interest the results disclosed by the Census of 1911 as contained in the recently published supplement to the seventy-fifth Annual Report of the Registrar-General of Births, Deaths, and Marriages in England and Wales.

The volume commences with a letter from the Registrar-General, Mr. Bernard Mallet, addressed to the President of the Local Government Board, in which it is pointed out that in the past the national life tables have been constructed in his office by members of his staff. In view of the fact, however, that the construction of life tables involves work of a highly technical character, it was determined on this occasion to obtain the advice of an actuary, and the services of Mr. George King were secured. The advantage of this expert advice is manifested in the excellent Report drawn up by Mr. King, which, with the life tables, forms the bulk of the volume.

### The History of Census-taking.

The first Census for Great Britain was effected in 1801. It was only after considerable opposition that the necessary powers for taking this Census were sanctioned by Parliament. The information was obtained, not from the heads of families, but from rectors, vicars, and overseers of the poor, and the final results were embodied in a single schedule giving particulars of the number of houses—inhabited and uninhabited—and of the number of persons—distinguishing the sexes—analysed according to three broad groups of occupations. A similar form was adopted in 1811, but on neither occasion was any attempt made to secure a return of the ages of the people. This analysis according to age did, however, find a place in the Census of 1821, though, curiously enough, it was again omitted from the enumeration of 1831. It was re-introduced in 1841, and has been an important feature in all subsequent enumerations. The first time that the schedules were filled in by householders was in 1851. By 1861 the importance of the Census, in combination with the records of births and deaths, as a means for measuring the progress of national vitality and for taking stock, as it were, of the results of public health reform, had come to be recognised. It was Dr. Farr who, in 1861, laid the foundations for the scientific analysis of the vital statistics of the nation, and, although the methods were imperfect, the basis was sound.

The Census alone merely furnishes an enumeration of the population on a particular day, and although it is true that by comparing the figures in districts, in occupations, in age groups, or in total with the corresponding figures of former Censuses, an idea can be obtained of the growth of the population, there are so many other factors—migration, for instance—which have to be taken into account, that the Census figures by themselves are inadequate to measure national vitality.

For this purpose it is necessary to make use of death returns, which, supplemented by the records of births, supply the means for the construction of life tables. The essential character of a life table is that it represents a stationary population unaffected by immigration on the one hand or emigration on the other. For the sake of simplicity it is assumed that it is possible to enumerate, say, 100,000 births, and the life table furnishes the number who survive year by year, out of this assumed number, to attain their first, second, third, and subsequent birthdays throughout life. It is, of course, impossible in point of fact to carry out any such operation on a sufficient number of births to render the figures of any value, but by an inverse process the life table is obtained from the actual records of the fluctuating population and the deaths that have occurred therefrom.

### What a Life Table Really is.

Let it be supposed that the data at hand are the Census returns of 1901 and 1911 relating to male lives in England and Wales, and the corresponding deaths in the interval. The figures are given in quinquennial groups of ages last birthday, 0-4, 5-9, 10-14, etc., throughout life, and the first operation is to find a suitable mean population, still retaining the figures in the original quinquennial groups of age. Various assumptions have been made for the calculation of the mean population. In the earliest tables it was supposed that for each age-group the arithmetical average would be an appropriate figure. Subsequently, on the principle that population begets population, it was assumed that the geometric mean for each age-group would be a better approximation. It was, however, found that the rates of progression in the various groups were not identical, and the assumption was, therefore, unsatisfactory in that the total of the mean populations in the groups did not correspond exactly with the total mean population calculated from the totals of the two Census enumerations. This difficulty has been overcome in the most recent tables by assuming that, while the total population increases in geometrical progression, the ratios which the groups bear to the total increase in arithmetical progression. This is, perhaps, a matter of technical detail, but it is mentioned as illustrating the care that has been expended on improving the methods of calculation which underlie the presentment of the tables.

Having, then, calculated the mean population, a further slight adjustment has to be made before the numbers living can be redistributed from the groups of ages to individual ages, to allow for the fact that the Census enumerations are not taken on

January 1, but on a date about three months later in the year. This being done, the figures resulting represent the numbers living in the original age-groups, as on January 1, in the middle of the period for which the death returns are available. Thus, on the basis of the Census enumerations in 1901 and 1911, the calculated mean populations refer to January 1, 1906. The next operation is the redistribution of the numbers living from the quinquennial groups of age to individual ages, and this was effected in the tables now presented by a mathematical process technically known as osculatory interpolation. It is unnecessary to enter into details here as to how this is actually carried out, though it may be remarked that the process is fully explained and illustrated in the Report.

A precisely similar operation is carried out with regard to the deaths. Thus the figures as so redistributed give a better presentment of the facts than if the data were extracted from the enumerations and death returns in individual ages. The reason is that the original data, both in the Censuses and death records, are affected by errors, ages being given in many cases at the nearest round number, in spite of all notices as to the necessity of accuracy.

The deaths, it will be observed, are the numbers that have occurred during a period of ten years, and when these are divided, age by age, by ten times the number living, as at the central point of time in the decennial interval, the results are the rates of mortality at successive ages which have been experienced in the period under review. With these rates it is at once possible to calculate from an assumed number of births the number of deaths that would occur before the attainment of the age of one year. Deducting these deaths, the result is the number of survivors on the first birthday, and by a similar continued process the numbers surviving on their successive birthdays in the supposed stationary population are calculated.

### Interesting Comparisons.

We are now in a position to appreciate the results that are disclosed by the life tables recently published. The tables are more numerous than usual, and, in fact, there are in all fifteen new tables.

The first tables are to be known as English Life Tables No. 7. They refer to males and females separately, and were constructed exactly as outlined above from the Census returns of 1901 and 1911 and the deaths in the decennial interval. The process of calculation was almost identical with that employed at the immediately preceding investigation, the results of which are embodied in English Life Tables No. 6, referring to the decennial period terminating on December 31, 1900. The figures are therefore strictly comparable in every way.

Two further new tables, referring respectively to males and females in England and Wales, should, however, be mentioned before the comparisons are instituted. These tables are based on the single Census enumeration of 1911, taken in conjunction with the deaths recorded in the three years 1910 to 1912. The principle of construction is the same, the only point of difference being that by reference to a single Census the effective date of comparison is brought down to July 1, 1911. These tables are to be known as English Life Tables No. 8.

One of the most effective comparisons that can be made of the results is shown in the following abridged tables, in which are given the numbers who die within five years out of 100,000, commencing at each age.

(*a*) Males.

| Age | No. 6 | No. 7 | No. 8 |
|---|---|---|---|
| 5 | 2,132 | 1,656 | 1,678 |
| 15 | 1,864 | 1,533 | 1,392 |
| 25 | 2,947 | 2,422 | 2,116 |
| 35 | 4,948 | 3,978 | 3,430 |
| 50 | 10,443 | 9,128 | 8,272 |
| 70 | 35,728 | 33,929 | 32,922 |
| 90 | 89,512 | 86,624 | 82,602 |

(*b*) Females.

| Age | No. 6 | No. 7 | No. 8 |
|---|---|---|---|
| 5 | 2,155 | 1,730 | 1,656 |
| 15 | 1,794 | 1,431 | 1,320 |
| 25 | 2,781 | 2,083 | 1,814 |
| 35 | 4,217 | 3,337 | 2,844 |
| 50 | 8,226 | 7,048 | 6,393 |
| 70 | 31,887 | 29,824 | 27,819 |
| 90 | 84,845 | 81,801 | 78,944 |

### State of England's Vitality.

With the single exception of the slight increase of the figure under Table No. 8 over Table No. 7 at age five in the male table, the figures throughout indicate a consistent and very material decline in mortality. It is, in fact, estimated that the improvement between the first and last tables is 19.04 per cent. for males and 21.96 per cent. for females.

This is an astonishing result, but the improvement is even more marked in the case of children under five years of age. For this section it was possible to compare the mortality for the three years 1910 to 1912 with the corresponding figures for ten years earlier—namely 1900 to 1902—and it appears that the improvement in the case of males is 25.61 per cent. and for females 26.19 per cent.

Here, then, is the strongest possible evidence of the utility of modern public health administration, and, so far from allowing the figures to act as a matter merely for congratulation, they surely provide an incentive for even greater efforts in the same direction in the future. If peace has her victories, she has her struggles also, and those who fancy that war alone can supply the stimulus mankind needs to nerve itself, may see and learn, from a study of the Registrar-General's figures, that so long as there is necessary work to be done, so long are our courage, tenacity, and brains provided with the opportunity for healthy struggle.

